# Hydrophobic pocket engineering of arylmalonate decarboxylase expands its substrate scope towards the synthesis of the (*R*)-enantiomers of sterically hindered carboxylic acids

**DOI:** 10.1039/d6cc02837c

**Published:** 2026-06-26

**Authors:** Elske van der Pol, Lisa-Marie Krammer, Johannes Eder, Dominik Gross, Roland C. Fischer, Kenji Miyamoto, Rolf Breinbauer, Robert Kourist

**Affiliations:** a Institute of Molecular Biotechnology, Graz University of Technology Petersgasse 14 8010 Graz Austria kourist@tugraz.at; b Institute of Organic Chemistry, Graz University of Technology Stremayrgasse 9 8010 Graz Austria; c Institute of Inorganic Chemistry, Graz University of Technology Stremayrgasse 9 8010 Graz Austria; d Department of Biosciences and Informatics, Keio University 3-14-1 Hiyoshi, Kohoku-ku Yokohama 223-8522 Japan

## Abstract

Hydrophobic pocket engineering of arylmalonate decarboxylase (AMDase) unlocked the synthesis of the (*R*)-enantiomers of enantiomerically pure α-aryl and α-alkenyl *n*-butanoic acids and one *n*-pentanoic acid.

## Abstract 2

Expansion of the AMDase substrate scope enables stereoselective access to bulky (*R*)-α-aryl and α-alkenyl butanoic acids with excellent enantiopurity.

## Graphical abstract

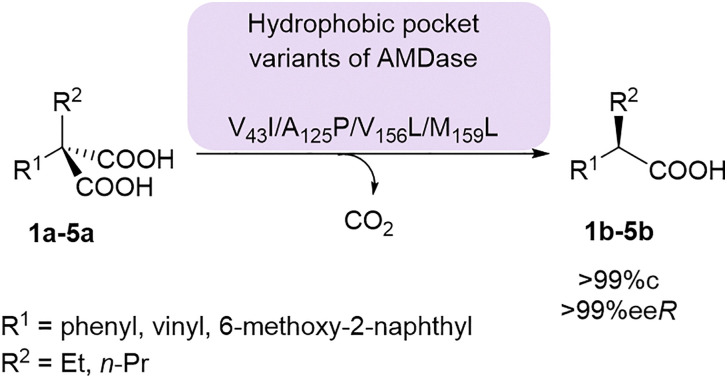

With their high selectivity and mild reaction conditions, enzymes are well established for the preparation of enantiomerically pure molecules.^1^ Limitations of the substrate scope of enzymes are a challenge, which can be addressed by enzyme engineering.^2,3^ The synthesis of α-substituted carboxylic acids is a typical example of the complementary strengths of chemical and enzyme catalysis. Direct alkylation of arylacetic acids using chiral auxiliaries is practicable and widely used.^4,5^ Recently, direct stereoselective alkylation of arylacetic acids using chiral lithium amide as a stereodirecting agent has been reported,^6^ giving access to a large number of α-alkyl carboxylic acids with enantiomeric excess ranging from 90 to 95%. A disadvantage is the requirement of the stoichiometric addition of an optically pure ligand. While the biocatalytic synthesis of α-chiral propanoic acids can be achieved by different enzyme classes with high to very high selectivity,^7–13^ there are only a few examples of enzymes producing the corresponding optically pure α-substituted butanoic acids (Table S1).^14–17^ Asymmetric decarboxylation of prochiral α,α-disubstituted malonic acids by bacterial arylmalonate decarboxylase (AMDase) gives access to α-aryl and α-alkenyl alkanoic acid derivatives such as non-steroidal anti-inflammatory drugs (NSAIDs)^18–22^ and α-heterocyclic propionic acids^23,24^ in high yield and excellent stereoselectivity.^20,25^ The stereoselectivity of the enzyme could be switched by transplanting the catalytic Cys-residue to the opposite side of the substrate^26^ and the activity of the resulting (*S*)-selective AMDase variant was improved by focused directed evolution,^18,21,25,27^ which has been explained by the formation of a second hydrophobic pocket in the active site.^28^

AMDase accepts an impressive number of substrates having different larger substituents, with the only requirement that they should bear a delocalized π-electron system required for the stabilization of the nascent charge upon decarboxylation. In contrast, the acceptance of the second substituent is very restricted. AMDase accepts malonic acids with a hydrogen atom, a methyl, a hydroxy, or an amine group, and halogen atoms.^29^ In their study on the first purification of the enzyme, Ohta and Miyamoto reported that 2-ethyl-2-phenyl malonate (1a) was inert to the enzyme.^30^ Recently, we showed that (*S*)-selective variants of AMDase having an extended hydrophobic pocket in the active site, such as AMDase V_43_I/G_74_C/A_125_P/V_156_L/M_159_L/C_188_G (‘AMDase ICPLLG’), accepted malonic acids with a larger second substituent and decarboxylated 2-ethyl-2-vinyl malonate (2a) to 2-ethylbut-3-enoic acid (2b).^31^ The excellent enantiomeric purity (>99% ee) of the product demonstrated the outstanding capacity of AMDase to discriminate between the similarly sized ethyl and vinyl substituents.

In contrast, wild-type AMDase did not convert 2a and showed only traces of 1b after 20 h (Fig. 1A).^31^ On the basis of this finding, we hypothesized that variants of the (*R*)-selective wild-type enzyme with an extended hydrophobic pocket could convert sterically hindered malonates with high stereoselectivity. Therefore, we prepared a series of α-aromatic and α-vinylic malonic acids with an ethyl and *n*-propyl group as the second α-substituent (1a–6a) and investigated the activity and stereoselectivity of (*R*)-selective AMDase IPLL (V_43_I/A_125_P/V_156_L/M_159_L) with amino acid substitutions in the active site hydrophobic pocket (Fig. 2A). Our results confirm that the increased size and hydrophobicity of the active site lead to a wider substrate spectrum, thereby enabling access to optically pure α-aryl and α-alkenyl *n*-butanoic and *n*-pentanoic acids (Fig. 1B).

**Fig. 1:**
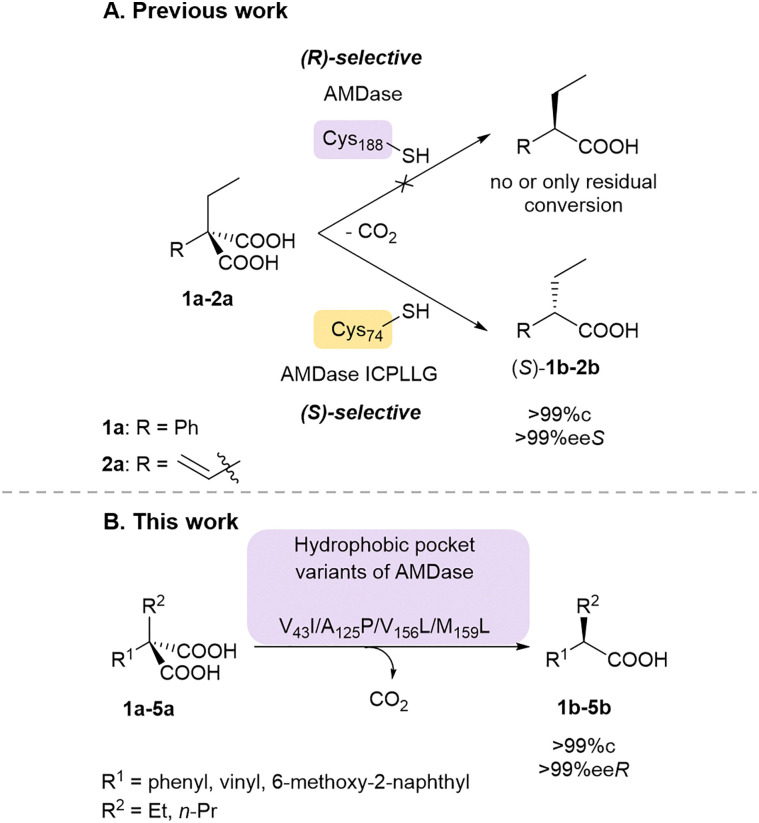
(A) Previous work: engineered (*S*)-selective AMDase accepts α-ethyl substrates, while (*R*)-selective AMDase WT does not. (B) This work: (*R*)-selective AMDase variants with an engineered hydrophobic pocket convert α-ethyl and *n*-propyl substrates.

**Fig. 2:**
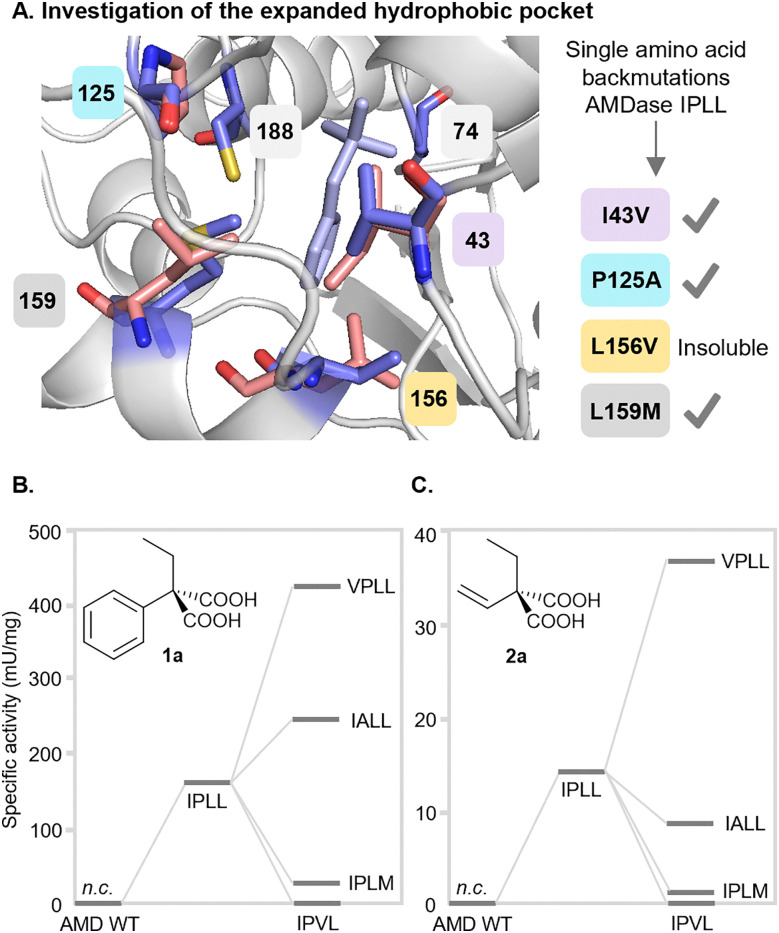
(A) Active site of AMDase, with altered amino acids highlighted in purple (wild-type, PDB entry 3IP8) and light pink (IPLL variant). (B) and (C) Specific activities of designed AMDase variants with substrates 1a–2a. AMDase IPVL produced a low level of soluble enzyme, and it was barely active. See Fig. S3 for details, *n* = 3.

In (*R*)-selective AMDase (C188), the mutations I_43_P_125_L_156_L_159_ increase activity towards aromatic α-methyl malonates.^21,25^ We were pleased to find that AMDase I_43_P_125_L_156_L_159_ also showed full conversion with 1a and 2a (Fig. 2B and C), producing (*R*)-1b and (*R*)-2b with high optical purity (>98% ee). To test the effect of these single substitutions, I_43_P_125_L_156_L_159_ was back mutated by single-point mutations, resulting in four AMDase variants: AMDase V_43_P_125_L_156_L_159_, I_43_A_125_L_156_L_159_, I_43_P_125_V_156_L_159_, and I_43_P_125_L_156_M_159_ (Fig. 2A).

The amino acid substitution M_159_L leads to higher activity towards α-methyl malonic acids (Fig. S1).^25,27^ Therefore, it is not surprising that I_43_P_125_L_156_L_159_ has also higher activity towards α-ethyl malonic acids than I_43_P_125_L_156_M_159_ (Fig. 2B and C). Unfortunately, soluble expression of AMD I_43_P_125_V_156_L_159_ was limited, and no specific activities could be measured for this variant. Reactions with the cell-free extract demonstrated poor conversion. All soluble variants retained the activity towards α-methyl malonic acids and achieved full conversion with 2-methyl-2-phenyl malonic acid 7a and 2-methyl-2-vinyl malonic acid 8a, respectively (Fig. S21–S33).

Variant I_43_A_125_L_156_L_159_ exhibits higher activity towards 1a than I_43_P_125_L_156_L_159_, whereas the activity towards 2a was insignificantly lower, showing that the contribution of this substitution has only a minor effect. Surprisingly, back-mutation of I43 to valine (AMDase V_43_P_125_L_156_L_159_) further boosted the activity for both α-ethyl malonates (Fig. 2B and C), resulting in the fastest (*R*)-selective AMDase variant for the conversion of 2a found so far (37.4 mU mg^−1^, 2.7-fold improvement). In the liganded structure of AMDase G_74_C/C_188_S, the distance between the C_β_-atom of V43 and the C_α_-atom of the ligand phenyl acetate is only 5 Å. Substitution of isoleucine by the smaller valine residue, lacking one methyl group, likely reduces steric congestion in this region and thereby facilitates more productive substrate positioning, resulting in enhanced reaction rates towards α-ethyl-malonates. Although *A*-values for methyl and ethyl are similar, van der Waals volumes increase substantially, indicating that flexible alkyl substituents may impose steric constraints.^32^

The results of the backmutation show that the substitutions V_156_L and M_159_L contribute the strongest to the activity of I_43_P_125_L_156_L_159_. At position 43, L and V are possible, and at position 125, A and P. Not surprisingly, α-aromatic malonates react faster than α-vinylic (Fig. S1), which has been observed before^25,31,33^ and can be attributed to the increased capacity to stabilize the nascent negative charge during the course of the reaction. For AMDase I_43_P_125_L_156_L_159_ and the faster AMDase V_43_P_125_L_156_L_159_, the measured optical purities are shown in Table 1. For (*R*)-1b, excellent ee's were found for both variants, while for (*R*)-2b, reduced selectivity was observed. This is surprising as the (*S*)-selective AMDase ICPLLG discriminates well between the two substituents of 2a (>99% ee). Back mutation of ICPLLG to AMDase VCPLLG resulted in a comparable positive trend in 2a (Fig. S1). However, for 1a, an adverse effect was observed. This can be rationalized by the altered binding mode of vinylic malonates compared to aryl malonates, in which the carboxylate is cleaved from the newly formed hydrophobic pocket.^19^ In this orientation, the residue at position 43 is part of the pocket accommodating the vinyl or ethyl substituent. Also, here, the substitution of isoleucine by the smaller valine residue reduces steric hindrance and allows for increasingly productive substrate positionings.

**Table 1:** Obtained enantiomeric excess (ee) of the chiral α-carboxylic acids produced by AMDase (on CFE), >99% conv. Aromatic acids 1b were derivatized to the corresponding methyl esters (Me-1b) using TMS-diazomethane. Experiments were performed in triplicate

AMDase	(*R*)-Me-1b	(*R*)-2b	(+)-3b	(*R*)-4b	5b^a^
IPLL	>99% ± 0.0	94% ± 0.8	77% ± 0.0	n.d.	n.d.
VPLL	>99% ± 0.0	96% ± 0.6	79% ± 0.7	>99% ± 0.0	>99% ± 0.0

a First eluting peak.

We then proceeded to investigate other substrates posing specific challenges for the enzyme. For the well-accepted α-ethyl substituted substrate 2a, we observed a 100-fold lower activity for both (*R*)- and (*S*)-selective AMDases compared to α-methyl-α-vinyl malonate (Fig. S1), which might be caused by steric reasons. To investigate the effect of the additional rotational degree of freedom, we examined 3a with bridged α-groups, limiting the free rotation of the alkyl side group (Scheme S1 and Fig. 3). Due to the conformationally restricted cyclohexenyl ring of 3a, it should be considered that the 2-cyclohexene malonate adopts a half-chair conformation with one carboxylate in the pseudoaxial position and one in the pseudoequatorial position.^34^ Typically, the axial substitutions are energetically less favored due to unfavorable interactions/steric clashes with other axial substituents and/or the p-orbitals of the unsaturated carbon–carbon bond. Therefore, bond cleavage of the α-carbon and the pseudoaxial carbonyl carbon would be favored. Subsequently, cleavage of the carboxylate introduces a partial negative charge on the α-carbon in the transition state. In contrast to 2a, where the vinyl group has free rotation, for 3a the partial negative charge can be stabilized through hyperconjugation of the aligned C=C bond. Both the conformational restrictions and the stabilizing effect would favor the acceptance of 3a over 2a.

**Fig. 3:**
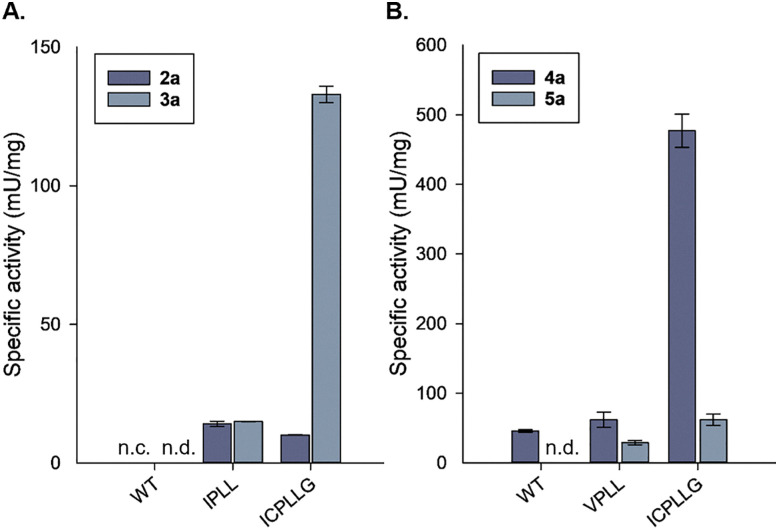
(A) Specific activities of AMDase variants with 2a and 3a. AMD WT does not convert 2a (n.c.). (B) Specific activities of AMDase variants with 4a and 5a. The conversion of 3a and 5a by AMD WT is too low to determine its specific activities (n.d.).

To investigate the impact of the G74C/C188G mutation on the specific activity, we determined the activity of the well-characterized (*R*)-selective AMDase IPLL and (*S*)-selective AMDase ICPLLG. For the AMDase IPLL, the rate for 3a was similar to that for 2a (approximately 15 mU mg^−1^). In contrast, AMDase ICPLLG showed a 13-fold higher rate when comparing the strained malonate 3a (133 mU mg^−1^) to α-ethyl malonate 2a (10 mU mg^−1^) (Fig. 3A). Previously, an isotope labeling study combined with QM/MM metadynamics indicated that for alkenylic substrates, the cleaved carboxylate, the α-carbon, and the cysteine would ideally be aligned at a 180-degree angle. While movement of the α-substituents is restricted for 3a, the reaction coordinates in AMDase IPLL are not aligned at the optimal 180-degree angle required for the borderline concerted mechanism of AMDase variants towards alkenylic substrates,^31^ which would clarify the similar rates observed for both substrates by AMDase IPLL.

In contrast, when 2a and 3a adopt the same binding mode as 2-methyl-2-vinyl malonate in AMDase ICPLLG, the α-carbon would be perfectly aligned at a 180-degree angle with the leaving carboxylate and C74. This binding mode allows the reaction to be borderline concerted, with the protonation by C74 being partially rate-limiting. The free rotation of the α-substituents, being hampered and not obstructing protonation, further facilitates improved reactivity. Together, these effects might provide an explanation for the observed enhancement in the reaction rates obtained for 2a and 3a by AMDase ICPLLG (Fig. 3A). The stereoselectivity of the two (*R*)-selective AMDase variants towards 3a was much lower than that for 2a. The small dialkyl malonates can bind in two inverse binding modes into the active site of AMDase, leading either to cleavage of the pro-*R* or the pro-*S* group, respectively.^31^ It can be easily imagined that due to the compact structure of 3a, both binding modes have similar binding energies, making their discrimination very challenging.

We then investigated the synthesis of both enantiomers of optically pure 4b, which has recently been described as an effective aldo–keto reductase 1C3 inhibitor.^35^ Variants VPLL (*R*) and ICPLLG (*S*) were chosen as they had shown the highest activity towards α-ethyl substituted malonates (Fig. 3B and 4). AMDase ICPLLG exhibited the highest specific activity toward 4a, while AMDase VPLL showed a ∼7-fold lower activity. Surprisingly, AMDase wild-type converted 4a, albeit with 10-fold lower activity than AMDase VPLL. In comparison, the situation is reversed for the less sterically demanding naproxen malonate, which was converted 5-fold faster by the wild-type than by AMDase ICPLLG.^18^ This difference in the activity of wild-type and variants towards differently substituted α-aryl-α-ethyl substrates underlines that the effect of substitutions in the active site hydrophobic pocket of AMDase is hard to predict and highly substrate-specific.

**Fig. 4:**
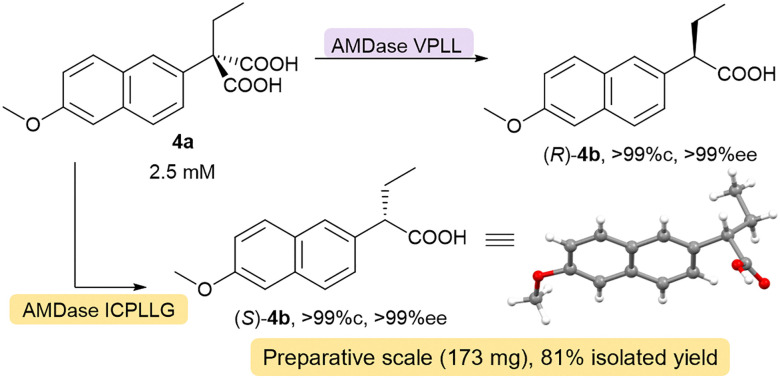
Conversion of 4a by AMDase VPLL and ICPLLG. (*S*)-4b was produced on a preparative scale using (*S*)-selective AMDase ICPLLG.

Furthermore, we explored the acceptance of propyl-(6-methoxy-2-naphthyl) (5a) and 2-propyl-2-vinyl malonate (6a). AMDase ICPLLG and VPLL indicated good acceptance of 5a (Fig. 3B), leading to 99% conversion. Yet the vinyl compound demonstrated only limited conversion (39% and 19% for AMDase ICPLLG and VPLL, respectively, Table S11). We attribute this to the less effective stabilization of the evolving negative charge by the vinylic compound, combined with the steric hindrance of the *n*-propyl substituent. For the AMDase variants VPLL and ICPLLG, the specific activities toward 5a were determined (Fig. 3B). Both variants exhibited markedly reduced rates for 5a compared to those for 4a, consistent with increased steric hindrance and greater rotational freedom of the *n*-propyl group. Overall, the engineered hydrophobic pocket of the variants ICPLLG and VPLL helps to overcome the limitations of the wild-type.

Both engineered AMDase variants were able to convert 4a and 5a with excellent enantioselectivity (>99% ee) (Table 1, Fig. S36 and S39), thereby providing access to both enantiomers of the aldo–keto reductase 1C3 inhibitor 4b. Recently, Adeniji and coworkers obtained the effective chiral acid 4b by synthesizing the racemate, followed by chiral HPLC separation.^35^ Since AMDase ICPLLG and VPLL demonstrated the ability to produce the chiral acid with high purity, an enzymatic step in the synthesis process could be advantageous and increase the theoretical yield from 50% to 100%. With two stereocomplementary enzymes in hand, we wanted to demonstrate their scale-up potential. Using the more active (*S*)-producing enzyme, we synthesized (*S*)-4b. The biotransformation with AMDase ICPLLG using 173 mg of 4a produced (*S*)-4b (Fig. 4) in 81% isolated yield. We confirmed the absolute stereochemical configuration of 4b obtained by AMDase ICPLLG-catalyzed decarboxylation *via* X-ray crystallography.

In conclusion, variation of active-site residues of AMDase, unlocked activity towards disubstituted malonic acids with an α-substituent larger than a methyl group, enabling the enzymatic synthesis of α-aryl and α-alkenyl *n*-butanoic acids and one example for an *n*-pentanoic acid with very high stereoselectivity. Point mutations in the hydrophobic pocket exert a strong and very substrate-specific effect on the conversion of these malonic acids, making it worth investigating small sets of hydrophobic pocket mutants for the conversion of sterically hindered malonic acids.

E. v. d. P. conceptualized, coordinated, performed experiments, visualized, and prepared the manuscript. L.-M. K. performed and analyzed the experiments with the naproxen malonates. She also prepared a part of the manuscript. J. E. generated the back-mutated AMDase variants and performed initial studies. D. G. performed the work-up and product purification of the preparative scale biocatalysis reactions and the crystallization. R. C. F. performed the X-ray diffraction and structure refinement. K. M. conceptualized and participated in discussions leading to the design of this study. R. B. conceptualized, provided funding, supervised, and prepared the manuscript. R. K. conceptualized, provided funding, supervised, visualized, and prepared the manuscript. All authors contributed to the data interpretation, reviewed, and approved the manuscript.

## Conflicts of interest

There are no conflicts to declare.

## Supplementary Material

CC-062-D6CC02837C-s001

CC-062-D6CC02837C-s002

## Data Availability

Supplementary information (SI): experimental details, sequences, NMR spectra, and (chiral) GC and HPLC chromatograms. See DOI: https://doi.org/10.1039/d6cc02837c. CCDC 2545010 contains the supplementary crystallographic data for this paper.^36^
